# Medical Items

**Published:** 1916-03

**Authors:** 


### MEDICAL ITEMS

### PUBLIC HEALTH REPORT OF THE SECRETARY OF THE TREASURY.

The annual report of the Secretary of the Treasury as it relates to the Public Health Service contains numerous recommendations bearing on the functions of that organization and evidences the great interest of this department in the extension and expansion of the governmental agencies for the protection of the public health.

In the development of general public health work, according to the Secretary, there is great need of additional medical officers. The number of requests for advice and assistance in health problems received from states and municipalities during the past year has far exceeded that in any similar period in the history of the Service, but the limited number of officers available for the work has prevented in many instances compliance with these requests.

The field investigations, the Secretary states, have served as a stimulus to state and local health agencies, and every effort should therefore be made to encourage and turn to practical account the interest in health matters awakened in the general public. For this reason an increase in the appropriation for field work is requested.

An additional building for the Hygienic Laboratory is urgently needed. The work of this institution has been greatly extended, particularly as it relates to the examination of viruses, serums and analogous products, a vast market for which has been recently created abroad. The safeguarding of these therapeutic agents requires great accuracy and precision and overcrowding is a serious handicap. In order that the public health may be better protected, an annual appropriation of $25,000 is recommended to be expended in carrying out the provisions of the law relating to the examination of these products.

The United States is the only government of importance which does not provide for the care and isolation of lepers. The establishment of a national leprosarium where the numerous lepers, most of whom are native born Americans, may be properly segregated and treated, thereby eliminating a menace to the health of others, is urged.

The further recommendations of the Secretary relate to the need of additional clerical assistance in order to meet the demands which are increasingly made on the Public Health Bureau.

---

### PITUITRIN IN UTERINE INERTIA.

Administered hypodermatically during the second stage of parturition (it should not be given during the first stage) Pituitrin is said to convert a case of tedious inertia into one of normal rhythmic labor, saving time, preventing suffering on the part of the mother, and diminishing the risk to the child which attends upon protracted labor. Furthermore in many cases it obviates the use of forceps.

Pituitrin is a pituitary extract, otherwise described as a solution of the active principle of the infundibular portion of the pituitary gland (in animals). This gland, as is well known, lies at the base of the brain.

There are a number of pituitary extracts on the market—products, it may be said in all fairness, not equal in therapeutic efficacy. To be of definite value as an oxytocic it is obvious that the solution or exract must be highly active. Owing to unavoidable variations in fresh glandular tissue, the amount of gland substance represented in the preparation is not an accurate index of its strength. Assurance of therapeutic activity can be obtained only by rigid assay. In view of these facts a recent statement of Parke, Davis & Co., with respect to their Pituitrin is peculiarly significant:

"Because of its importance in obstetrical practice we have given much attention to a determination of the proper strength and standardization of Pituitrin. The result of our investigations is a product of high potency, representing the average activity of 0.2 gramme of fresh posterior pituitary lobe to each cc. of the solution. As an oxytocic Pituitrin stands without a rival. There is no more active pituitary extract. Pituitrin is standardized by the two accepted methods of determining pituitary activity; the blood pressure test and the oxytocic test, the latter by use of the isolated uterus. Every lot of Pituitrin represents the same high degree of activity."

Pituitrin is supplied in glaseptic ampoules of 1 Cc. and 1-2 Cc. capacity, convenient for hypodermatic administration.

---

### ABOUT SOPORIFICS.

The measure of a soporific's usefulness is found not in any single quality but in its adaptability to the various nervous and mental conditions encountered in daily practice. Potency, although it is the first essential, cannot be taken as a standard by which to judge a remedy of this class. Danger either of immediate evil effect or habit-forming too often accompanies this quality. The sphere of usefulness of such remedies is limited to cases which are under the constant supervision of the physician or trained attendant· It is esential to have a safe sedative-hypnotic for the vast majority of cases. Such a remedy must be free from habit-forming drugs and dangerous heart depressants. On the other hand it must possess sufficient potency to relieve all nervous manifestations and highly excited mental states not absolutely requiring the administration of an opiate. It must be a remedy in which dependence can be placed not only as to positive therapeutic effect, but also as to freedom from deleterious results.

These requirements are fully met in Neurosine. Neurosine is a potent sedative-hypnotic-antispasmodic not dependent on habit-forming drugs or coal tar derivatives for its therapeutic effect. It possesses positive sedative power and this power is made available wherever desired by absolute freedom from danger. This combination of maximum potency and perfect safety with the added advantage of exemption from narcotic laws make Neurosine the most satisfactory soporific for routine use.

